# Exploring the Experiences of West African Immigrants Living with Type 2 Diabetes in the UK

**DOI:** 10.3390/ijerph16193516

**Published:** 2019-09-20

**Authors:** Folashade Alloh, Ann Hemingway, Angela Turner-Wilson

**Affiliations:** Department of Public Health and Human Sciences, Faculty of Health and Social Sciences, Bournemouth University, Bournemouth BH1 3LH, UK; aheming@Bournemouth.ac.uk (A.H.); aturnerwilson@bournemouth.ac.uk (A.T.-W.)

**Keywords:** diabetes management, immigrants, dietary habits

## Abstract

The increasing prevalence and poorer management of Type 2 diabetes among West African immigrants in the UK is a public health concern. This research explored the experiences of West African immigrants in the management of Type 2 diabetes in the UK using a constructivist grounded theory approach. In-depth individual interviews were conducted with thirty-four West African immigrants living with Type 2 diabetes in the London area. Fifteen male and nineteen female adult West African immigrants with age range from 33–82 years participated in the study. Participants were recruited from five diabetes support groups and community settings. Initial, focused and theoretical coding, constant comparison and memos were used to analyse collected data. Three concepts emerged: Changing dietary habits composed of participants’ experiences in meeting dietary recommendations, improving physical activity concerned with the experience of reduced physical activity since moving to the UK and striving to adapt which focus on the impact of migration changes in living with Type 2 diabetes in the UK. These address challenges that West African immigrants experience in the management of Type 2 diabetes in the UK. The findings of this research provide a better understanding of the influencing factors and can be used to improve the support provided for West Africans living with Type 2 diabetes in the UK, presenting a deeper understanding of socio-cultural factors that contribute to supporting individuals from this population.

## 1. Introduction

Diabetes Mellitus (DM) disproportionately affect immigrants when compared to the general population in the United Kingdom (UK) [1,2]. This is due to the health inequality reported among immigrant populations in the UK [1]. Higher prevalence of Type 2 Diabetes Mellitus (T2DM) has been reported among Africans than the general population in the UK [3].

The prevalence of DM has been on the increase in the UK. In the late 1990s, only 2% of the UK population were living with DM and less than 5% of the health budget was spent on DM and its complications [4]. Currently, Public Health England (PHE) reported the prevalence of DM has increased to 6.8% [5]. In addition, it is estimated that 15–16% of all deaths in England occur because of DM and its complications [6]. Complications due to DM account for one in five cases of all cardiovascular admissions in the UK [7], making the disease an important public health concern. It is estimated that the UK spends over £23 billion on DM both directly and indirectly. The direct financial burden of DM to the economy is estimated to be £9.8 billion and about £13.9 billion as indirect cost [8,9]. More than 75,000 deaths are associated with DM in the UK, exceeding the expected mortality rate for such condition by more than 24,000 deaths [10]. DM is increasingly a public health concern because of the health inequality posed by the condition in the UK [1]. There are several types of DM, however, this study only focuses on T2DM as it accounts for over 90% of all DM cases [11].

Studies have reported the contributions of environmental and lifestyle factors to the genetic factors that predispose Africans immigrants to increase the development of T2DM and other non-communicable diseases [12]. Furthermore, evidence linking immigration and lifestyle changes and T2DM prevalence among African immigrants was found in the United States of America [13]. The study showed higher insulin secretion and resistance among African American than Africans in Africa. This shows the impact of lifestyle differences due to change in environment can lead to the development of T2DM through alteration of insulin secretion and sensitivity. This study will focus on exploring T2DM management among West African immigrants (WAIs) due to the increasing population of this group in the UK [14]. This study aims to understand the experiences of WAIs living with T2DM can contribute to supporting this population with managing their T2DM condition.

### Ethnicity and Type 2 Diabetes

Reports of the high prevalence of T2DM among WAIs in the UK have been recorded [3,15]. T2DM prevalence increases with years of migration among WAIs [16]. WAIs are three times more at risk of developing T2DM than the general population [17,18,19]. This suggests that immigration has an impact on the health of immigrants and contributes to the higher prevalence of T2DM this population. Furthermore, there are health disparities among ethnic minorities compared to the general population in the UK [20,21,22]. Much can be learned from the accumulated health experiences of immigrants in countries with high immigrant population such as the UK [23].

This follows the National Institute for Health and Care Excellence (NICE) guidelines recommendations on the need for more research on barriers and facilitating factors in the management of T2DM among immigrants [24]. Although studies have reported factors that contribute to T2DM management, they focused on access to healthcare services, adherence to care services and patient-provider relationship [25,26]. This has left a gap for studies that qualitatively explore the experiences of WAIs living with T2DM in the UK.

This study is designed to better understand the experiences of WAIs living with diabetes in the UK. Findings from this research are anticipated to provide a better understanding of the diabetes management process among WAIs. This will help to raise awareness of the challenges that WAIs experience in managing T2DM due to changes in lifestyle prior to migration [27]. The aim of this study is to provide insights into the challenges that WAIs experience in managing T2DM in the UK.

## 2. Materials and Methods

A qualitative research approach has been selected for this study as it was found to be the most suitable to meet the aim of this study. Specifically, Constructivist Grounded Theory (CGT) seeks to develop explanatory theory from meanings and interactions with others in social processes about a phenomenon [28,29]. CGT is described as a methodology that focuses on the social interaction of the phenomenon [30]. T2DM management is not well explored and understood in terms of the challenges experienced by WAIs in the UK. Adopting CGT is seen as a methodology that can help in addressing the exploration of T2DM management among WAIs in the UK. The CGT approach places emphasis on diverse worlds and multiple realities and the complexity of specific world, views and actions. Charmaz [31] places emphases on the views, beliefs, values, assumptions and feelings of individuals that are conducting the study. In the case of this study, CGT was adopted as it is suitable for the aim of this study, which is to understand the experiences of WAIs in managing T2DM in the UK.

### 2.1. Participant Selection

**Figure 1:**
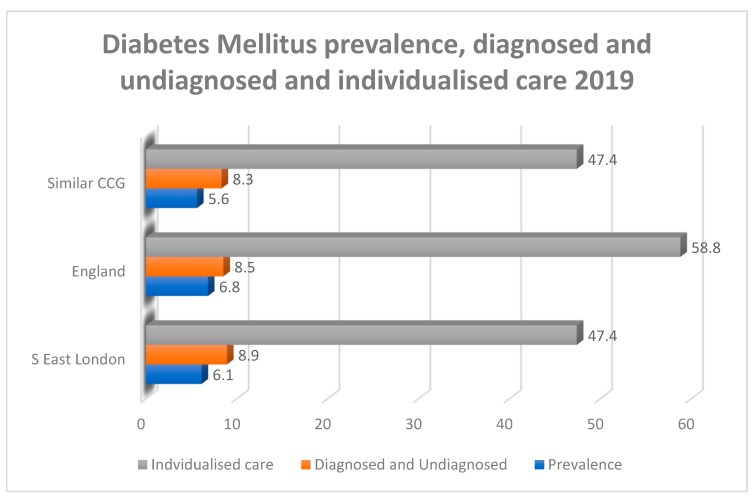
Mellitus prevalence in South East London compared to England Adapted and modified from: Public Health England [5].

Individuals living with T2DM and originally migrated from West Africa (WA) to the UK were recruited for this study. The data collection was carried out in London borough councils. London was found to be a suitable location for data collection for two main reasons. First, according to the London Strategic Clinical Networks (LSCN), there is a high population of WAIs in the London area [32]. Second, there is a high prevalence of T2DM among residents of London of more than 475,000 [32,33]. T2DM prevalence in London ranges from 3.5% to 8.2% compared to 6.8% for the rest of England (Figure 1).

Five support group sites were selected and contacted for access to recruiting members for this study. In three sites, meetings were held every month while two sites hold meetings once in two months. Support groups meet at least once in a month to provide information and support for members. In addition, churches and mosques were approached for the opportunity to recruit their members in this research (Table 1). Participants were accessed at these locations through the head of the organisations who acted as gatekeepers for recruitment. Recruitment commenced from March 2017 and was concluded in March 2018.

A combination of purposive and snowball sampling techniques were adopted to recruit WAIs living with T2DM in the UK. These were used as initial sampling techniques as expected in CGT [31,34]. A theoretical sampling technique was then used to recruit individuals that have information to contribute to the emerging concepts as the study progressed. For example, theoretical sampling required recruitment of individuals that had support with dietary needs and those that live alone, this is to better understand the influence of support in dietary habits of WAIs.

In total thirty-four qualitative interviews were conducted which is adequate for this study as saturation was achieved at this point. This point was achieved when no new insights emerged from participants’ narrations of managing T2DM in the UK.

### 2.2. Interview Procedure

Semi-structured interviews were carried out with each participant. An interview guide was designed to direct the flow of the conversation (Supplementary 1). The interview guide was reviewed by all authors (FA, AH and ATW).

The guide focused on exploring:

- The experiences of managing T2DM in the UK.
- The influence of living in WA in the management of T2DM in the UK.
- The impact of environmental influence in the management of T2DM in the UK.

A pilot study session was organised with three WAIs prior to the commencement of the main study. This helped to review the interview guide to include exploring influences of living in WA.

In-depth interviews were carried out with all thirty-four participants. Each interview session lasted an average of an hour, but two sessions ran for over two hours. Each interview took place in quiet places such as meeting rooms in support group premises, and homes of participants. The interviews were conducted by FA, while AH and ATW reviewed the interview transcripts after each interview. Disagreements were discussed and reviewed before the next sets of interviews.

The interview was conducted in English as all participants were able to communicate in English. All interviews were audio-recorded and all interviews were transcribed. In addition to the interviews carried out, other sources of data were included in the data collection process (Table 2).

### 2.3. Data Analysis

Interview transcripts were then analysed with the broad concept of CGT using a bottom-up inductive approach. Following CGT according to Charmaz [35], the method allows participants to tell their stories in their own terms. To support the analysis process, a computer software package was used in this study. Specifically, Nvivo 11 by QSR International (QSR international Pty Ltd. 2018) [36] was used to assist with the data analysis and management process. Initial, focused and theoretical coding in CGT were employed to analyse all transcripts [37]. Constant comparisons were done within data from one interview and then between different interviews. Similar concepts that seem related to the same category were brought together to form a category using line-by-line and in-vivo coding. Thus, the bottom-up approach helped to identify codes that formed concepts and finally categories. To ensure the credibility of the analysis process, each interview coding was carried out by FA, second and third authors (AH and ATW) independently reviewed the analysis. Differences in analysis were discussed and agreed in research meetings. As expected in CGT approach, data collection and analysis were carried out simultaneously [37]. Interviews are arranged in a set of 3–4 within a period, data analysis carried out on the interviews and emerging patterns incorporated in interview discussions for next sets of interviews. In addition to interview analysis, observation was used as source of data (Table 2). For example, observational data were gathered during the data collection at the support groups. Also we observed the food preparation of some participants in their home. These multiple data sources gave context to the data analysis by complementing emerging findings from transcript analysis.

Memos were written as the analysis progressed to move beyond description to the conceptualisation of participants’ experiences [31]. Memos and field notes written during the interviews were used to inform the analysis process. Questions were asked to raise the analysis beyond description to the conceptualisation level, these include:

- How do West Africans come to experience living with Type 2 diabetes mellitus in the UK?
- What do they make of their experiences?
- What are the important factors that influence the process of managing Type 2 diabetes mellitus in the UK?

Each transcript was revisited and reread thoroughly and then compared with the codes identified from the initial coding. As coding progresses, analysis sensitivity tends more towards how individuals perceive living with T2DM and the effect of assumptions about what is known and the influences of their condition on their lifestyle. Using these assumptions allows for understanding the actual influences of living with T2DM in the daily lifestyle. Using constants comparison, codes were revisited and refined to further develop the categories identified in the transcripts.

### 2.4. Ethics

The ethical application for this study was received on 9 January 2017 with a formal letter on 11 January 2017 from Bournemouth University ethical committee (Approval number 13441). All participants read and signed the consent form before data collection. To ensure confidentiality, all identifying information was removed from participant responses and pseudonym was assigned to all participants.

## 3. Findings

Nineteen females and fifteen males participated in the interviews for the study. These individuals met the inclusion criteria to be recruited for participation in this study as they migrate from WA and living with T2DM. No participant withdrew after consenting to participate in the study. Participants’ age ranges from 33 years for the youngest participant to 84 years for the oldest participant (see Table 3). The categories identified in this study were from participants’ responses, which followed the principles of the CGT approach [37]. Each supporting quote was labelled with the participant’s gender and age to provide a context for participants’ narration.

### 3.1. Challenges in Managing Type 2 Diabetes Mellitus in the UK

This section presents the findings of the study in relation to issues associated with challenges and dealing with changes in the management of T2DM in the UK. It illustrates the management of T2DM as a challenging process that affect their dietary and physical activity experiences while living in the UK.

#### 3.1.1. Changing Dietary Habits

The changing dietary habits experienced influence their decisions on food choices, and the impact on managing T2DM. Participants discussed how they had to make efforts on changing their dietary habits after diagnosis. This was a particularly important aspect of T2DM management as a result of the influence of self-management in the process.

“I was advised to stop eating so many of the foodstuffs that I was used to eat, as we were told they had too much starch”.

(Ken 48 years, male)

The changes experienced in living with T2DM in the UK are mainly due to differences in knowledge and perception of healthy dietary habits from living in WA. This highlights the importance of understanding of healthy and unhealthy dietary perception to WAIs.

#### 3.1.2. Understanding Unhealthy Diets

Participants discussed what they understand as unhealthy diet and how this can impact their management of T2DM. This is interesting as many refer to refined sugar content of diet as the man unhealthy aspect of meals in the UK. Different aspects of changing influence dietary habits; sugar content was a very important aspect of eating healthy to participants interviewed.

“Obviously refined sugars are not good but sugars that are absorbed very slowly into the body like starches, starch based foods are to be preferred to sugar while foods that yield sugar content readily like grapes should be avoided”.

(Konge 45 years, male)

Interestingly, most participants discussed how they tried to avoid sugar in their diet since being diagnosed. This highlights their concern about refined sugar and food with refined sugar. However, there was limited knowledge about natural occurring sugar in food or high caloric foods.

“I didn’t have a sweet tooth as such like cakes and chocolates and all that even before the diagnosis of diabetes”.

(Bobaro 57 years, male)

In their efforts to continue managing T2DM, they have to start paying attention to the food that they eat since being diagnosed. However, the attention seems to change with their change of environment from WA to the UK. In WA, their attention is mainly concerned with avoiding refined sugar while little or no attention is paid to natural sugar contents in foods. For example, participants discussed how they were advised to avoid sugar, but they still had diets high in carbohydrate.

“There were specific aspects of shopping that I found interesting, it meant changes to approaches to grocery shopping. Yes so I would pay a lot of attention to packages and nutritional information and it was quite an eye opener finding out how much sugar is in stuffs, that was a valuable experience I had”.

(Bolula 48 years, female)

and:

“Yes, just a simple illustration coming from Nigeria, West African we have sugar only in teas and coffees but not in anything else, so I arrive in the UK and there is sugar in everything else expect in teas”.

(Nimbabo 49 years, male)

This highlights dietary misconception in regards to impact of sugar in T2DM management. There is a need to provide adequate information concerning natural sugar in diet and contribution to T2DM management among this population.

#### 3.1.3. Having Large Dietary Portions

Following the discussion of food content, having to understand moderation is another issue that was also well discussed among the participants. It is particularly of importance in relation to dietary portions. Most participants talked about having recommendations to reduce their food portion. This has been challenging as they reported being familiar with having large food portion. Understanding moderation in healthy eating is more challenging and harder to meet management recommendations.

“You know how we like our food, I have large food portion unlike this small things that they eat here, it just cannot be enough for me that is really frustrating”.

(Mange 57 years, male)

and:

“I used to try my best but when I have to eat all the vegetables to get full because they digest quickly as far as I am concerned, what more can i do? These does not fill me up like my eba and fufu7 does” (Kinjile 72 years, male) (eba and fufu are popular West African diets derived from cassava tuber. Cassava is a root tuber plant that is rich in important nutrients and starch and so may have health benefits when consumed in moderation).

There seems to be less attention about dietary moderation among participants recruited within community settings when compared with those from support groups.

“I eat when am hungry for food, the issue is that I have big appetite and so their small food sizes cannot be enough for an African man like me. I have to focus on me when am hungry is the thing”.

(Mange 57 years, male)

#### 3.1.4. Paying for Healthy Diets

In addition to preparation difficulty, inadequate time, unfamiliarity with food type, cost of healthy food ingredients and the difficulty of not having familiar food types were also identified as contributing to struggling with healthy living.

“Yes it is because when I switched to the healthy food, anytime I go for shopping, I spend a lot of money and am thinking is it because it is not Nigerian food. At times that I used to buy a lot of Nigerian food and like that but when it comes to all this healthy food, even small portion is a lot of money”.

(Gamboe 42 years, male)

#### 3.1.5. Getting Dietary Support

Getting support with food was an essential aspect of striving to adapt among participants. Male participants particularly discussed how they require help to prepare healthy meals from close family members such as partners and close female friends. This helps them to stay on healthier dietary habits.

“And my wife who is not also very well herself, so many years after she still makes special effort to make sure that I have my meals even at time when have other constraints I will struggle to do it myself and she has tried to make extra effort to make sure that I eat and so that have been good. She has been monitoring and just keeping an eye on what I eat”.

(Zurisa 69 years, male)

and:

“I have got someone to help me but she kind of like English food, she is a Nigerian friend of mine but she doesn’t even know anything about Nigeria. She hasn’t been there but she helps me a lot in healthy eating before I even detect that I have got type 2 diabetes. So am good in eating healthy food, so when I got to know that I have to be checking out what am taking and all that, she helps me a little bit more”.

(Dee 33 years, male)

In addition to support with dietary habits to manage T2DM, participants also discussed easier access, better efficient services and free use of healthcare services in the UK than WA which contributes to management of their condition.

“I have always gone for yearly medical check-up, I just feel it is good as the hospital is there anyway”.

(Yaranto 67 years, female)

In general, there is a perception of better healthcare services in the UK than in WA, which contributes to their management of T2DM. Participants discussed how they found the treatment they receive to be very helpful in the UK. Mainly because most hospitals are better equipped with both work force and excellent standard medical equipment than what is found in Africa where human and financial resources are limited.

“Although I will rather talk to doctors that can fully understand my concerns. I would say it is better to know that I will be treated with the best available facilities here which are not available in Africa”.

(Bolu 44 years, female)

Another interesting aspect of challenges experienced in living with T2DM among WAIs in the UK is the struggle to meet physical activity recommendations by healthcare professionals. This is particularly difficult for participants diagnosed with T2DM in WA.

### 3.2. Meeting Physical Activity Needs

In addition to the dietary challenges in managing T2DM as presented above, participants discuss the challenges of meeting physical activity recommendations in the UK. All participants interviewed in the study acknowledge the need to increase their physical activity level. Physical activity level was discussed to have reduced significantly since moving to the UK.

“The kind of life we live here is just difficult to meet those exercise that my doctors ask me to always get, you know? I mean I go to work which helps a bit but this place just seem to have made everything within my reach o, i hardly need to walk or do any major work is the issue here”.

(Josera 42 years, male)

#### 3.2.1. Comorbidity Impact on Meeting Physical Activity Needs

Some other participants highlighted comorbidity as the reason for their reduced physical activity. This made increasing their physical activity level as recommended by healthcare personnel to be very difficult to achieve as they make efforts to meet up with the recommendations.

“Well sadly I cannot afford gym but there was a time when just getting by a day was a problem but I a lot of time I just resting lying down but after sometimes I started walking short distances by increasing you know that walk until you know I could go round the block…you know sometimes it will take me 15 to 20 mins when I started initially when I started to rest as I walked long because my heart which is the effect of my illness and my heart.”.

(Bobaro 57 years, male)

and:

“Well, I can’t even walk as I have arthritis, I have knee replacement surgeries so mobility is one of my biggest problems. I would love to walk but I struggle to walk but from time to time when I can… once a week or two or if I can at all… I go to the gym to use the treadmill, because at least I can hold on to something because I have mobility issue as well as diabetes. You know my bad arthritis, so that is all I do. I don’t do anything much”.

(Mam 64 years, female)

#### 3.2.2. Environmental Influence on Physical Activity

While in the narration of others, it was highlighted that the environmental influence in achieving daily tasks easily has contributed to their reduced level of physical activity.

“Physical activity? This is not really something I get involved in now, it has been all too difficult to get enough exercise since I came here even before I got my diabetes. I think it has to do with the values attached to going to the gym among us as Africans. I cannot just be going to gym or running as exercise, it is not part of me”.

(Wemi 56 years, female)

#### 3.2.3. Finding Alternative Treatment

The impact of belief was noted in finding alternative treatments. This is particularly among those that were interviewed within community settings; they highlight some of the treatments they engaged in to manage their condition. They believe some of these herbs can cure their T2DM. Although participants mentioned herbal medication use after moving to the UK, it was mainly in relation to when they have complications especially after many years of living with T2DM.

“I had a stroke once as a result of complications from my diabetes, I was told there is no help that they give you in hospital. Curing the stroke was entirely up to my own efforts. I was fortunate to be in Africa then, you know we have herbs. I was given herbs which I will rub on my right side and sneeze like snuff and sneeze my head off which they say activate my blood circulation”.

(Egbede 64 years, male)

Finding alternative treatment for their T2DM or complications that arise from the condition is seen as the impact of their belief in these alternative treatments. Hoping for a cure for T2DM or the complications of T2DM enhances the use of alternative treatment for this population. Majority of the participants expressed the hope that a cure for T2DM is found as this is what really interests them. They mention having a cure can take away their burden of living with the disease. This contributes to their using of alternative medications like herbs that were claimed to cure T2DM.

“I was in a desperate state at this point and was willing to do anything to cure my diabetes. I drank my own urine, drank ewuro (ewuro (*Vernonia amygdalina*) is also known as bitter leaf), ate onions and garlic but no cure”.

(Yeriyaya 77 years, male)

and:

“In Africa, we were told to eat bitter food as they help cure or at least reduce the blood sugar, so I used to eat aloe vera, ewuro and green igba” Green Igba is Unripe garden egg (Solanum aethiopicum).

(Bolula 48 years, female)

### 3.3. Striving to Adapt

Participants referred to striving in their health as something that they have not attained. The findings from the analysis of participants’ narrations referenced to the challenges that they encounter after migration to the UK. This finding is in line with studies on immigrants that are living with T2DM in Western countries were striving to adapt to challenges have affected T2DM management [3,38]. Participants recounted their management of T2DM in the UK as challenging mainly due to changes that were experienced which differ from their prior experiences in WA.

“The stress is not easy o, crazy something is that the changes that I have to deal with are not the same”.

(Bobaro 57 years, male)

and:

“We know things will not be the same as we used to have, the issue is how to adapt to these changes. As you know, some of the things are easier while others are difficult for us”.

(Orisa 52 years, male)

**Figure 2:**
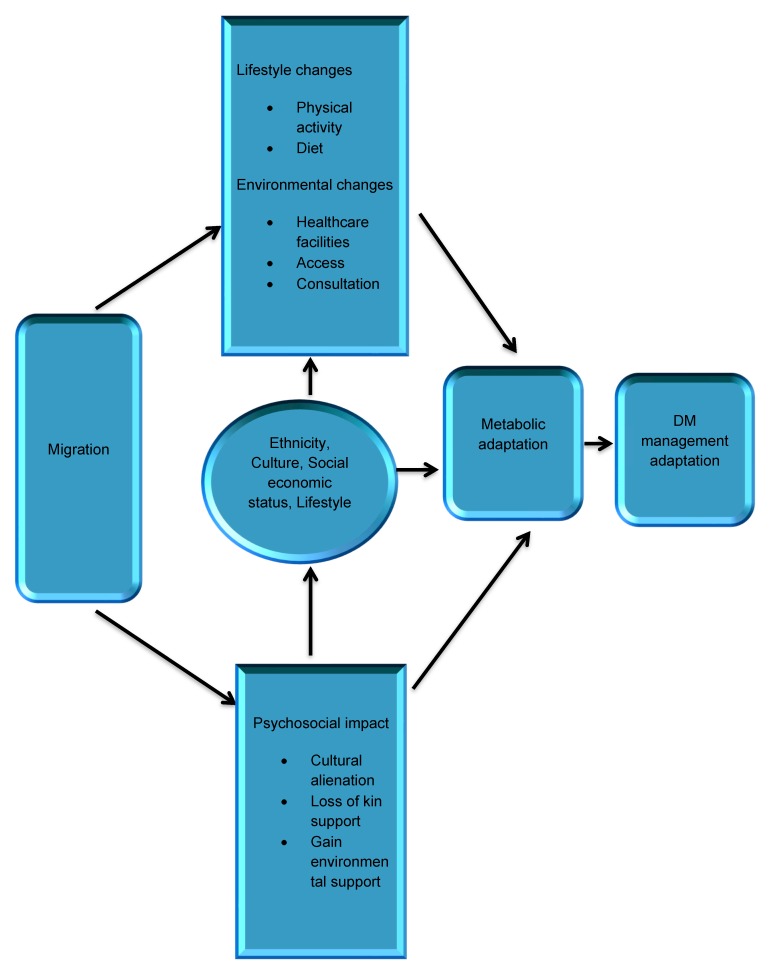
Effect of Migration on Type 2 Diabetes Mellitus Management. Adapted and modified from Misra and Ganda [39].

Striving to adapt highlights the discussions of participants on the changes that are experienced as a result of managing T2DM after migration. They gave accounts of their efforts in achieving the recommended healthy living since being diagnosed with T2DM. How they have worked on changing some of the contributing factors in their life to help them in the management process. The findings of this study highlight important aspects of T2DM management among WAIs as influenced by migration to the UK (Figure 2).

## 4. Discussions

The empirical aim of this research is to better understand the experiences of WAIs in living with T2DM in the UK. This study is in light of the political climate in the UK, the decision to leave the European Union will influence the population diversity in the country [40]. Understanding T2DM among WAIs who are among the fastest growing ethnic group is important for public health planning and interventions. The findings of this study suggest that WAIs experience daily challenges that affect their T2DM management. These challenges are mainly concerning the struggles in adapting to management recommendations in the UK. Adaptation struggles were mainly pronounced with lifestyle choices, which are changes in dietary habits which suggest differences in dietary habits prior to migration. The other aspect is improving physical activity, which highlights the reduction in the physical activity level of WAIs after migration to the UK affecting T2DM management. The finding that WAIs are faced with challenging situations in reconciling T2DM management recommendations in the UK with their lifestyle of living before to migration is interesting. The complex interplay of factors has been reported to affect the level of physical activity amongst black and ethnic minorities in the UK [41]. The effect of changing environment due to migration was highlighted as influential in T2DM management process [42,43].

Adapting to living with chronic disease has been suggested in the literature as an influencing factor in the management of health conditions [44]. Adaptation is defined as the process of thinking and feeling that individuals’ conscious awareness in making choices affects their human and environmental integration [45]. Adaption is a lifelong process [46]. This study found that striving to adapt encompasses the processes in which WAIs develop an awareness of their needs and desire in the management of T2DM. Maslow [47] explained that needs and desire are the two motivational states that affect humans in meeting their goals. Needs are classified into five motivational needs. These five states of needs can be divided into basic needs such as physiological, safety, love and esteem while the higher state of need is growth need such as self-actualisation [48]

Maslow’s paper on the theory of human motivation has the lower-order need of basic human need (physiological and safety needs). Basic needs are linked to survival purposes needs (food, sleep, sex, shelter, clothing, safety and security). The higher level of needs as explained by Maslow [47] is the self-actualisation need which is mainly linked to life experiences or achieving a status where humans become all they can be intellectually and creatively.

Maslow stated that people are motivated to attain or fulfil certain needs [47]. The fulfilment of basic needs (lower level) leads to seeking the self-actualisation (next level). Basic needs or lower-level needs have to be met before higher-level growth needs can be fulfilled [48,49]. Concerning the findings of this study, striving to adapt is a form of basic needs that WAIs work on fulfilling. This is particularly about basic needs such as food, drink and shelter challenges. The fulfilment of these needs is dependent on lived experiences as it is concerned with why people act and think as they do. Adapting to the challenges they face in managing T2DM in the UK is a form of need that they aspire to meet. For example, participants expressed their efforts to meet basic needs such as diet, physical activity that are similar to Maslow’s lower-level need.

In meeting the needs required for adaptation of WAIs in managing T2DM in the UK, support is needed. T2DM management is a chronic condition that requires input from individuals, families and society [50,51]. Maslow again recognises the need for receiving care and be cared for as a form of need being met [52]. Meeting the needs of managing T2DM is essential and requires social support in achieving the adaptation needs that participants strive to achieve. This is particularly pronounced in the limited support from friends and family found among immigrants [53]. Participants expressed their need for support, particularly among male participants as this helps in managing their condition. Migration affects the continuity of relationships with members of the kin network [54]. This can reduce contacts with kin networks, which affect the support gotten in achieving a healthier lifestyle [55]. This, in turn, affects the adaptation process in the management of T2DM in the UK. In this study, reports on the reduced kin network support were highlighted as an important aspect that affects the management of T2DM. The advantage of having kin network support was noted with reports on aspects such as healthy food preparation, reminders about physical activity and social activities. These aspects of T2DM management are important as they influence their efforts in striving to adapt to the management process.

An important aspect of this study finding is the cost of managing T2DM as reported by participants. This is concerning evidence that individuals with low socioeconomic status have worse glycaemic control than those with higher socioeconomic status [56]. African immigrants are reported to be disadvantaged in terms of socioeconomic status [57]. Individuals in low-income household are reported to lower expenditure on fruit and vegetables than higher-income household [58]. This is important giving the influence of socioeconomic status in the management of T2DM, there is a need for policy to support the economic cost of managing T2DM especially among immigrant population. For example, free or subsidised gym membership and incentives for these practices can be implemented. Also, support of healthy habits such as fruit and vegetable subsidy or maximising household income for low-income household especially those living with long term condition can be implemented.

Furthermore, these individuals work on finding a balance to integrate their preferred dietary and lifestyle practices prior to migration into the management process in the UK. Understanding the influences of lived experiences before migration on T2DM management can help in ensuring that an acceptable balance is found for meeting both basic and higher-level needs. This finding is supported by a qualitative study that explored the dietary practice of Ghanaian immigrants in the UK [59]. The study reported that, although there were influences of migration in the dietary preference of immigrant populations, there was still the preference for traditional diets that are seen to be familiar. These present important implications for nutritionist and dieticians working with individuals from West Africa. Majority of dietary recommendations and guidelines are derived from Western countries [60]. There is a need for research into dietary recommendations that are based on ethnic group dietary habits. The dietary challenges that WAIs experience as found in this study shows the need for research and building capacity among immigrant ethnic groups with low incomes. These can be important for Nutritionist working with individuals from ethnic groups.

By holding on to beliefs about the impact of healthy dietary and lifestyle choices on the control of blood glucose, participants were able to adapt some lifestyle choices. It is important to understand that beliefs are influenced by lived experiences. This has been reported in the literature confirming this study’s finding [61].

### 4.1. Cultural Influence on Type 2 Diabetes Mellitus Management

Culture is seen as an important aspect that relates to different aspects of this study. Throughout the findings of this study, cultural influence has been emphasised by participants. It is, therefore, important to further explore the concept of culture as related to the findings of this study. Culture is defined as the fundamental system of organisation of individuals that is designed to ensure survival, provide common ways to find meaning and purpose of life [62]. The cultural system encompasses the beliefs, values and lifestyle used in successfully adapting to the environment.

Culture is seen to go beyond shared beliefs and understandings but includes the practices that are because of those beliefs and understandings [63,64]. Culture is an essential part of the way of life as it shapes the beliefs, understanding, practices and acts of individuals or groups of individuals. These attributes of culture can determine the survival of individuals, which highlights the importance of culture in every aspect of human life.

The impact of culture and lifestyle practices of Africans living in Africa can help our understanding of T2DM management among WAIs in the UK. In terms of healthy eating, although there was a report of positive behaviour change after T2DM diagnosis, there are misconceptions and knowledge gap on what constitutes healthy eating [65,66]. Similarly, physical activity level was reported to be poor, this was because of limited knowledge on how physical activity impacts glycaemic control. Furthermore, brisk walking is the most common form of physical activity among Africans living in Africa [67]. Furthermore, studies have reported poor self-monitor of blood glucose level among people living with T2DM in Africa [68,69,70]. For example, less than 15% of people living with T2DM are able to test their blood glucose level at home [71]. Similarly, medication adherence was generally low in studies, T2DM doctors reported non-compliance of most patients to pharmacotherapy as issues with working with individuals living with T2DM in Africa [72]. The low adherence to pharmacotherapy might be due to the high importance placed on alternative medicines. People living with T2DM in Africa associate T2DM treatment with medical facilities but seek T2DM cure from traditional healers [73,74,75]. In general, there is poor management of T2DM among people living with the condition in Africa. This might explain some of the lifestyle practices that continue to contribute to the management of T2DM among WAIs in the UK.

Differences in cultural settings within each society also influence the management of T2DM as found in this study. In WA, the practice of collectivism is prominent in societies; this explains the collectivist influence of societal norms and practices in the perception of WAIs towards the management of T2DM. While in the UK, the individualism of UK society encourages lone/individual management of T2DM. In changing environments from WA to the UK, the change in culturally collective society to an individualistic society can be challenging to adapt to living. The transition from a collectivist to an individualistic society can be challenging for WAIs due to differences in the cultural dynamic of both societies. This can be complicated because of living with T2DM.

Understanding the dynamics of cultural influences on health can contribute to better supporting these individuals to achieve better management of T2DM. As found in this study, the impact of culture was seen in all aspects of T2DM management among WAIs. The actions, beliefs and experiences of these individuals are influenced by their culture, which invariably contributes to their willingness to adapt to changes required to manage T2DM in the UK.

### 4.2. Limitations and Strengths

This research explored the experiences of WAIs in managing T2DM in the UK. To our knowledge, this is the first research that has attempted to understand the management of T2DM among WAIs in the UK using a constructivist approach. Further studies are needed to confirm the concerns that were found in managing T2DM in this study. Initially, a purposive sampling technique was used to recruit participants. This sampling technique might have led to unknown selection bias, the use of multiple sampling techniques due to the challenges of recruiting from this population may affect the transferability of the findings. Caution needs to be taken when applying the findings from this study to a more generalised context in the management of T2DM among other people living with T2DM in the UK. However, CGT offers the potential for theoretical generalisation to wider and similar contexts and informing statistical studies to further findings [76]. The findings from this study can help in understanding the challenges faced by other immigrant groups in managing their health conditions. For example, the impact of lived experience prior to migration as identified in this study can be useful in understanding the health challenges of immigrant populations.

Apart from Nigeria, Ghana, Sierra Leone and Gambia that are English speaking countries in WA, other countries in the region are French-speaking countries. Therefore conducting this study in English might have excluded individuals from French-speaking countries in West African region. However, the majority of immigrant senders to the UK are from English speaking countries due to commonality in communication language. In addition, the listed countries are higher senders of immigrants to the UK compared to the French-speaking countries. For example, Nigeria is among the top ten senders of immigrants from Africa to the UK [14]. Hence it is not anticipated that English language use has excluded individuals with useful contributions to this study.

## 5. Conclusions

This study revealed that WAIs living with T2DM can have different experiences for their management regime from the general population due to migration from WA to the UK. Challenges in meeting T2DM management recommendations were found to be related to lifestyle changes in the UK. The findings from this study are important when considering interventions to help these individuals with managing their T2DM condition. This is because there is a projection of an increase in the population of WAIs in the UK and the poorer management outcomes experienced by this population may increase the burden of T2DM in the UK. Policymakers should consider the challenges experienced by this population, particularly in reducing the health inequalities by formulating policies to improve T2DM among WAIs and the general population. In conclusion, we argue that poor management of T2DM among WAIs can be associated with lifestyle changes that are experienced due to change in environment from WA to the UK. Supporting individuals from this group in making informed lifestyle choices concerning useful information to healthy living will be valuable in promoting the health of WAIs living with T2DM in the UK.

## Figures and Tables

**Table 1:** Recruitment venues.

Study Sites	Location	Interaction	Participants Recruited
Diabetes management support groups	London area	Presentation by researcher	24
Community	Church	Discussions with congregation	7
	Mosque	Imam introduced researcher to potential participants	3

**Table 2:** Data sources.

Data	Type of Data	Sources
Primary data	Interview Observation Conversation	Participants’ response Support group observation Daily living activities Religious places Participants’ photos/albums
Secondary data	Literature	Electronic literature as data Food time-table Photo/album Hospital appointments and consultations

**Table 3:** Demographic features of participants.

	Female, *n* = 19	Male, *n* = 15
Age		
Range (years)	40–69	33–82
Employment		
Employed	12	6
Retired/out of job	7	9
Education		
Tertiary	12	7
Secondary	3	5
Primary	2	3
Place of diagnosis		
UK	6	5
West Africa	13	10
Nationality		
Nigeria	13	9
Ghana	5	3
Gambia	1	3

## Supplementary Materials

The following are available online at https://www.mdpi.com/1660-4601/16/19/3516/s1, Supplementary 1: Grounded theory Semi structured interview prompts focused on how participants live with diabetes.
