# Supplementary material for: Exploring the Experiences of West African Immigrants Living with Type 2 Diabetes in the UK
Source: Int J Environ Res Public Health. 2019 Sep 20;16(19):3516. doi: 10.3390/ijerph16193516 (PMC6801552; doi:10.3390/ijerph16193516)
Supplement: Supplementary file 1 [file ijerph-16-03516-s001.pdf]

Grounded theory Semi structured interview prompts focused on how participants live with diabetes.

Overarching question

Can you tell me about your experiences of living with diabetes in the UK?

Initial Open-ended Questions

Can you tell me about when you were diagnosed with diabetes?

Where were you when you during the diagnosis, UK or Africa?

How was it for you to be diagnosed with diabetes? If you can recall, what did you thinking about it?

How has your lifestyle changed after your diagnosis?

Can you describe the changes you experienced?

How did you manage the condition?

Intermediate Questions

Has the management of diabetes changed the way you live in the UK?

How has your lifestyle changed since your migration to the UK?

Do you think there are barriers to your managing your diabetes in the UK?

Are there supports to assist in the management of your diabetes in the UK?

Are there other ways that you would like to improve your management of your diabetes in the UK?

What is the worst (Negative) experiences you have had in managing your diabetes in the UK?

What is the best (Positive) experiences you had in managing your diabetes in the UK?

What will be your ideal way to manage diabetes in the UK?

What are the useful things in helping you managing your diabetes in the UK?

Ending questions

What are the things you think are valuable to manage diabetes well?

What advice would you give someone that is newly diagnosed?

Is there something that you might not have thought about before that occurred to you during this interview?

Is there anything else you think I should know to help me better understand your experiences?

Is there anything you would like to ask me?

Not all questions will be asked and they may not be asked in chronological order. In addition, it will be dependent on the participant answers for further question.

In this stage, no interruption once the participants start to discuss their story of managing diabetes, notes will be taking for further questioning after they have finished relating their experience of managing diabetes.
